# Survey of Haemosporidian Parasites in Wild Stone-Curlews (*Burhinus oedicnemus*) in the Canary Islands: First Molecular and Histopathological Evidence of *Leucocytozoon* sp. Infection

**DOI:** 10.3390/ani15233381

**Published:** 2025-11-22

**Authors:** Ana Colom-Rivero, Antonio Fernández, Lucía Marrero-Ponce, Raiden Grandía-Guzmán, Lucía Caballero-Hernández, Candela Rivero-Herrera, Cristian M. Suárez-Santana, Eva Sierra

**Affiliations:** Unit of Veterinary Histology and Pathology, University Institute of Animal Health and Food Safety (IUSA), Veterinary School, University of Las Palmas de Gran Canaria (ULPGC), 35413 Las Palmas de Gran Canaria, Canary Islands, Spain; antonio.fernandez@ulpgc.es (A.F.); raiden.grandia@gmail.com (R.G.-G.);

**Keywords:** *Cytochrome b* gene, lineage CIAE02, megalomeronts, skin, *Avipoxvirus*, *Aspergillus fumigatus*

## Abstract

Avian haemosporidians are globally distributed protozoan parasites transmitted by hematophagous vectors, yet information on their occurrence in the Stone-curlew (*Burhinus oedicnemus*), particularly from the Canary Islands, is scarce. Between 2020 and 2024, 47 Stone-curlews were examined for *Haemoproteus*, *Plasmodium*, and *Leucocytozoon* spp. using nested PCR targeting the *cytochrome b* gene, followed by sequencing and phylogenetic analysis. Histopathological examination was performed on formalin-fixed tissues. *Leucocytozoon* sp. DNA was detected in one individual (case FS415/23), with identical sequences amplified from multiple organs. Phylogenetic analysis placed this isolate within lineage CIAE02, previously reported in raptors and other avian taxa. Microscopic evaluation revealed megalomeronts in the liver, kidney, and skin, consistent with *Leucocytozoon* infection. Despite concurrent infection with *Avipoxvirus* and *Aspergillus fumigatus*, no prominent inflammatory reaction surrounded the haemosporidian tissue states. The only prior haemosporidian reported in *Burhinus* is *Haemoproteus burhinus*, described from *B. oedicnemus saharae* in Iraq, and no *Leucocytozoon* infections have previously been recorded in this genus. Therefore, this represents the first evidence of *Leucocytozoon* infection in the Stone-curlew, extending the known host range of lineage CIAE02. These findings highlight the relevance of integrative diagnostic approaches for detecting latent or cryptic haemosporidian infections in non-passerine avian hosts.

## Simple Summary

Haemosporidian parasites are vector-borne protozoans infecting a wide range of birds and occasionally causing disease in susceptible species. However, information on these parasites in the Stone-curlew (*Burhinus oedicnemus*), especially in the Canary Islands, is lacking. In this study, 47 wild Stone-curlews were examined using molecular and histopathological methods to detect these parasites. We report the first molecular and histopathological confirmation of *Leucocytozoon* sp. infection in this species. Parasite DNA was found in several organs, and characteristic tissue stages were identified in the liver, kidney, and skin. Phylogenetic analysis revealed that the detected lineage (CIAE02) has previously been described in raptors and other birds. These findings expand the known host range of *Leucocytozoon* CIAE02 and emphasize the importance of the combined use of molecular and histopathological tools to uncover hidden parasite diversity in wild avifauna.

## 1. Introduction

Hemoparasites are vector-borne protozoans with a cosmopolitan distribution that infect a broad range of vertebrate hosts. Among birds, the most frequently reported are avian haemosporidian (order Haemosporida, phylum Apicomplexa), with more than 250 species classified into the three most common avian blood parasite genera: *Haemoproteus*, *Plasmodium*, and *Leucocytozoon* [1,2]. These blood parasites are transmitted by hematophagous dipterans (*Haemoproteus* by louse flies or biting midges, *Leucocytozoon* by black flies or biting midges, and *Plasmodium* by mosquitoes) [3,4,5,6,7].

The life cycle of avian haemosporidians is complex, involving both vertebrate and invertebrate hosts. It includes three principal stages: (i) an exo-erythrocytic phase (merogony or schizogony) phase, during which asexual replication occurs within the tissues of the intermediate vertebrate host; (ii) an erythrocytic stage, where gametocytes develop within blood cells and become infectious to hematophagous insect vectors; and (iii) sexual and sporogonic phases within the dipteran definitive host vector, where fertilization and sporozoite formation occur. Mature sporozoites are subsequently transmitted to new avian hosts during vector blood meals [8,9].

Molecular approaches have markedly enhanced the accuracy of diagnosing avian haemosporidians. In particular, mitochondrial DNA primers targeting the *cytochrome b* gene have enabled the reliable detection and identification of *Haemoproteus*, *Plasmodium*, and *Leucocytozoon* species across diverse avian hosts and regions. These molecular tools have overcome many of the limitations of traditional microscopic methods, particularly in cases of low parasitemia or mixed infections [10].

However, recent research has revealed a greater diversity of avian haemosporidians than historically recognized, highlighting the need for further investigations to resolve their phylogenetic relationships [1,11,12,13,14,15,16]. Sequencing remains essential to achieve fine-scale taxonomic resolution. Only through the analysis of sequence data can parasite lineages be accurately identified, cryptic diversity detected, and morphologically similar but genetically divergent species distinguished [3,17].

The genetic diversity within the order Haemosporida is substantial, with over 5312 different lineages currently listed in the MalAvi database [18] is no longer accessible through its former public URL, we used an updated version of the database provided directly by one of the authors (Javier Pérez-Tris, pers. comm.) (accessed on 3 July 2025). Nevertheless, the existence of distinct genetic lineages does not necessarily imply that all represent valid species. Establishing species boundaries requires integrative analysis employing multiple genetic markers and morphological characterization [18]. Addressing this taxonomic uncertainty remains challenging, largely due to the difficulty of sampling diverse avian hosts across extensive geographic ranges [5].

Beyond their taxonomic and genetic diversity, avian haemosporidians are of considerable pathological importance. Infection outcomes vary widely, ranging from subclinical infections to severe disease, depending on the parasite species, host susceptibility, and infection intensity [3,9]. Common pathological manifestations include anemia, hepatosplenomegaly, vascular damage, and multifocal necrosis in parenchymatous organs. Exo-erythrocytic stages, particularly the large megalomeronts of *Leucocytozoon* spp., can cause extensive tissue destruction, inflammation, and hemorrhage, occasionally leading to mortality in susceptible hosts [3,9].

In addition, abortive development of haemosporidian parasites is increasingly recognized as a relevant source of pathology. In non-compatible or accidental hosts, parasites may initiate development but fail to complete their life cycle, resulting in the formation of atypical exo-erythrocytic stages [3,9,19]. Although such infections do not produce circulating gametocytes, they may induce severe tissue damage and are often underdiagnosed when relying solely on blood smears.

The Stone-curlew (*Burhinus oedicnemus*) (order Charadriiformes, family Burhinidae) is a nocturnal wader of conservation concern, widely distributed across Europe, North Africa, and parts of Asia, and represented in the Canary Islands by two endemic subspecies: *B. o. insularum* (eastern islands) and *B. o. distinctus* (western islands) [20,21,22,23,24,25,26]. As a species adapted to steppe environments, the Stone-curlew is particularly sensitive to global environmental change and is regarded as both a flagship and an umbrella species, since conservation efforts directed toward its protection also benefit other fauna associated with steppe habitats and contribute to the maintenance of these ecosystems [26,27]. In the Canary Islands, the species inhabits open, arid environments such as coastal plains, scrublands, and cultivated fields, typically nesting directly on the ground among sparse vegetation. It primarily feeds on insects, other invertebrates, and small vertebrates, exhibiting crepuscular and nocturnal activity patterns that reduce competition and predation risk. The Stone-curlew is largely sedentary within the archipelago, although some local movements occur in response to food availability and climatic conditions [23]. Both Canarian subspecies are legally protected due to their limited distribution and ecological vulnerability [20,21,22]; however, their populations continue to face threats, primarily from anthropogenic factors, such as collisions with power lines, as well as from natural causes, including infectious and parasitic diseases [28,29,30,31,32,33]. Given their restricted range and conservation concern [34], a thorough understanding of infectious agents, including blood parasites, viruses, and fungi, is essential. The present study aims to determine the presence of avian hemosporidia in the Canary Islands Stone-curlew and discuss the potential implications of these findings for the conservation of the endemic subspecies.

## 2. Materials and Methods

### 2.1. Study Area and Climate

**Figure 1:**
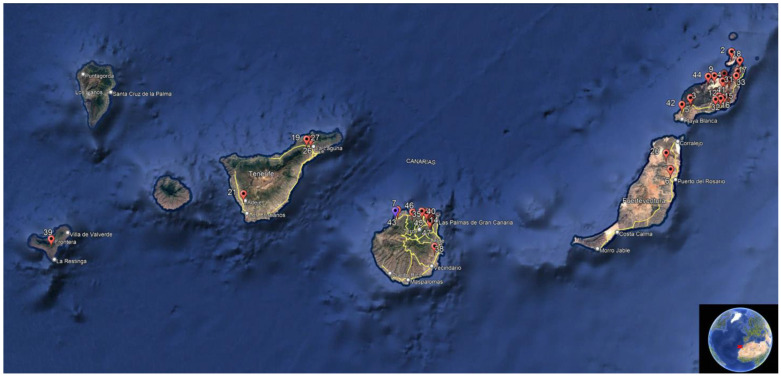
Map of the Canary Islands showing the locations where the birds included in the study were found. The positive *Leucocytozoon* case is indicated. (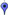 Positive case).

The Canary Islands are a volcanic archipelago located off the northwestern coast of Africa (27°37′–29°25′ N, 13°20′ and 18°10′ W) (Figure 1), composed of seven main islands that vary in topography and climate. The region has a predominantly subtropical climate characterized by mild temperatures throughout the year, low annual rainfall, and the influence of the northeast trade winds. Rainfall is irregular and mainly concentrated between November and March, while the summer months are typically dry. The eastern islands (Lanzarote and Fuerteventura) are more arid and dominated by steppe and semi-desert habitats, whereas the western islands (Tenerife, La Palma, La Gomera, and El Hierro) are more humid, with higher altitudes and greater vegetation cover [https://www3.gobiernodecanarias.org/ (accessed on 10 November 2025)] [https://www.gevic.net/index.php (accessed on 10 November 2025)].

Figure 1 shows the locations where the specimens included in this study were found. Geographical coordinates are provided in Table A1 in some cases, the coordinates are approximate depending on the precision of the location site.

### 2.2. Birds and Tissue Sampling

The stone-curlew is distributed across the Canary Islands with an estimated 1000–2500 resident pairs. Population densities are highest in the eastern islands, with 485–3972 individuals in Lanzarote and 278–1351 in Fuerteventura, and smaller populations in La Graciosa (7–59 individuals) and Alegranza (~10 pairs). In contrast, the western islands host fewer breeding pairs: approximately 250 in Gran Canaria, 50–60 in Tenerife, 100 in El Hierro, and around 20 and 12 pairs in La Gomera and La Palma, respectively [35].

A total of 47 deceased Stone-curlews were collected between 2020 and 2024 through the Canarian Wildlife Health Surveillance Network, a governmental program established to investigate wildlife mortality in the Canary Islands (Red Vigía Canarias; Order No. 134/2020, 26 May 2020). Carcasses were submitted for necropsy at the University Institute of Animal Health and Food Safety (IUSA), University of Las Palmas de Gran Canaria (ULPGC), and selected for this study based on their decomposition status following the veterinary pathology protocol of the IUSA; only specimens categorized as state: 1 (very fresh), 2 (fresh), or 3 (incipient decomposition) were included, while those in advanced (state 4) or very advanced decomposition (state 5) were excluded. Biological information, including age, sex, date of carcass discovery, and body condition (assessed following previously established protocols [29,36,37]), is provided in Table A1.

All necropsies were performed using standardized and consistent procedures [38], which include a thorough macroscopic assessment of each carcass and photographic documentation of all organs and any detected lesions. Tissue samples were obtained from major organs, such as the liver, lungs, kidneys, and intestines, as well as from any skin areas exhibiting lesions (Table A1). Sterile, individually wrapped swabs without transport medium (Vircell S.L., Granada, Spain) were used to collect samples from the oropharyngeal cavity, cloaca, coelomic cavity, and brain, as previously described [28,39,40]. All biological material was archived at the IUSA. Fresh, unfixed tissue was stored at −80 °C for subsequent molecular analyses, whereas additional samples were fixed in 10% buffered formalin for histopathological examination.

In cases where sex, age, or body condition could not be determined, this limitation was primarily attributed to the absence of suitable tissue resulting from the cause of death, such as trauma from vehicle collisions, or to the lack of macroscopically identifiable organs, such as the gonads, which prevented sample collection for histopathological examination (Table A1).

### 2.3. Molecular Analysis of Avian Hemosporidia

For each animal, small tissue samples from the lung, liver, and kidney were pooled into a single composite sample using a sterile swab, except in one case where only a lung tissue sample was available. These samples were placed in tubes containing viral transport medium with HEPES buffer, gelatine, bovine serum albumin, sucrose, and compatible antibiotics to preserve pathogen stability and viability during transport and storage (Transport Medium for the Collection and Preservation of Viruses, Chlamydia, and Mycoplasma, Vircell S.L., Granada, Spain). Skin samples were mechanically homogenized in DNA/RNA Shield. For molecular extraction, a 100 μL aliquot of each diluted pooled sample was combined with an equal volume of DNA/RNA Shield, while 200 μL of the macerated skin sample was used after centrifugation.

A simultaneous DNA/RNA extraction was conducted using a magnetic bead-based method on an automated robotic platform, following the manufacturer’s protocol for the ZYMO DNA/RNA extraction kit (ZYMO Research, Freiburg, Germany). To validate the extraction process, both a negative control (nuclease-free water) and a positive control (an *Avipoxvirus* positive sample previously confirmed in our laboratory) were included [29].

A total of forty-six pooled samples were analyzed, comprising tissue swabs from liver, kidney, and lung. In one bird, only a lung sample was available. In cases where pooled samples tested positive, individual tissue samples were subsequently analyzed, including those originally included in the pool as well as additional organs that showed structures compatible with haemosporidian infection in the histopathological study (e.g., skin) (Table A1). To detect avian haemosporidian parasites (*Haemoproteus*, *Plasmodium*, and *Leucocytozoon*), conventional polymerase chain reaction (PCR) was used following a nested PCR protocol previously described by Hellgren et al. (2004) [17], which targets the *cytochrome b* gene of the mitochondrial genome. Specifically, DNA templates (2 μL) were amplified in a 12.5 μL reaction mixture containing 1× PCR buffer with MgCl_2_ (2.5 mM), 0.4 μM of each primer, 0.2 mM of each deoxynucleotide triphosphate (dNTP), 0.05 U/μL of Taq DNA polymerase (Roche Applied Science, Vienna, Austria), and diethylpyrocarbonate (DEPC)-treated water. Primary PCRs were conducted with an initial denaturation at 94 °C for 3 min, followed by 20 cycles of 94 °C for 30 s, 50 °C for 30 s, and 72 °C for 45 s, with a final extension at 72 °C for 10 min. Secondary PCRs were performed under the same conditions, except that 35 cycles were applied instead of 20. The external PCR employed the primers HaemNFI (5′-CATATATTAAGAGAAITATGGAG-3′; where “I” denotes the universal base, inosine) and HaemNR3 (5′-ATAGAAAGATAAGAAATACCATTC-3′). For the internal PCR, the primers HaemF and HaemR2 were used to amplify *Plasmodium* spp. and *Haemoproteus* spp., while HaemFL (59-ATGGTGTTTTAGATACTTACATT-39) and HaemR2L (5′-CATTATCTGGATGAGATAATGGIGC-3′) were used for *Leucocytozoon* spp.

PCR products (5 μL) were analyzed by horizontal electrophoresis on a 2% agarose gel stained with GelRed^®^ (Biotium, Inc., Fremont, CA, USA). Diethylpyrocarbonate (DPEC)-treated water was employed as the negative control in both PCR assays, while a commercially available purified DNA of *Plasmodium falciparum* (Vircell S.L. Ref.: MBC148-R) was used as the positive control. Successful amplification of *Leucocytozoon* spp was indicated by PCR products of 478 base pairs in length (bp), excluding primer sequences, and 480 bp for *Haemoproteus* spp. and *Plasmodium* spp.

### 2.4. Detection and Sequencing of PCR Products and Phylogenetic Analysis

As the PCR assay amplifies fragments of nearly identical size for *Plasmodium*, *Haemoproteus*, and *Leucocytozoon* spp., the specific genus could not be determined based solely on the electrophoretic band pattern. Therefore, amplicons obtained from conventional PCR [17] of positive samples were purified using the Real Clean spin kit (REAL, Valencia, Spain) and subsequently subjected to bidirectional Sanger sequencing. Sequencing was performed using the product of the second internal PCR from the semi-nested PCR, employing primers HaemF, HaemR2, for *Plasmodium* spp. and *Haemoproteus* spp. and HaemFL, HaemR3L for Leucocytozoon spp. The resulting nucleotide sequences were analyzed both internally and by comparison with publicly available sequences in GenBank through BLASTN (Basic Local Alignment Search Tool) (+ 2.17.0 version) analysis on 16 September 2025 and MalAvi database lineage name [18] consultation on 3 July 2025. Multiple sequence alignments of Plasmodium spp., *Haemoproteus* spp. and *Leucocytozoon* spp. sequences were carried out using ClustalW.

Phylogenetic and molecular evolutionary analyses were performed using MEGA version 12 [41], incorporating 21 nucleotide sequences retrieved from GenBank, 19 of *Leucocytozoon* spp., and two *Plasmodium* spp. sequences as the outgroup. Phylogenetic trees were constructed using the Neighbor-Joining (NJ) and Maximum Parsimony (MP) algorithms based on pairwise genetic distances estimated by the Maximum Composite Likelihood (MCL) method according to the Tamura (1992) [42] model. The rate model allowed for 59.10% of sites to be evolutionarily invariable (I). The analysis included 22 nucleotide sequences, with 526 aligned positions in the final dataset. The topology robustness was assessed using a bootstrap consensus tree based on 1000 replicates. Branches supported by fewer than 50% of bootstrap replicates collapsed, and bootstrap values (expressed as percentages) were displayed next to the corresponding branches [42].

### 2.5. Histopathological Examination

Formalin-fixed tissue samples were routinely processed for histopathological evaluation. Samples were dehydrated through graded alcohols, cleared in xylene, and embedded in paraffin wax. Three µm-thick sections were stained with hematoxylin and eosin (H&E) following standard protocols and examined under a light microscope (Olympus BX51, Olympus Corp., Tokyo, Japan). Although all available tissues were processed, histopathological results are presented only for the individual that tested PCR-positive for haemosporidian infection. The evaluation focused on identifying tissue alterations associated with haemosporidian infection, including the presence, localization, and morphology of exo-erythrocytic stages (meronts and megalomeronts), inflammatory responses, necrosis, and vascular lesions. Special attention was given to the detection of abortive developmental stages in non-erythrocytic tissues, as well as the presence of intra-erythrocytic gametocytes. Representative parasitic stages were photodocumented using a digital microscope camera (DP21) and a 0.5X (U-TV0.5XC-3) adapter (Olympus Corp., Tokyo, Japan).

## 3. Results

### 3.1. Birds and Tissue Sampling

The sample included representatives of both Stone-curlew subspecies present in the Canary Islands. Of these, 22 individuals (47%) belonged to *B. o. insularum* (20 from Lanzarote and 2 from Fuerteventura), while the remaining 25 birds (53%) corresponded to *B. o. distinctus* (19 from Gran Canaria, 5 from Tenerife, and 1 from El Hierro). Of the 47 Stone-curlews examined, 20 were identified as female (20/47), 19 as male (19/47), and the sex of 8 individuals (8/47) could not be determined. In terms of age distribution, 27 birds were adults (27/47), 18 were juveniles (18/47), and 1 was a chick (1/47), with the age of 1 bird being undetermined (1/47). Body condition assessment revealed that 14 (14/47) individuals were cachectic (grade 1), 18 (18/47) were underweight (grade 2), and 13 were in an ideal stage (grade 3). In 2 cases, the body condition could not be evaluated. Full biological information for each specimen is provided in Table A1.

### 3.2. Detection and Sequencing of PCR Products

A fragment of the mitochondrial *cytochrome b* gene consistent with Haemosporida parasites was initially detected in the pooled tissue sample (liver, kidney, and lung) and later confirmed in the corresponding individual tissues, as well as the skin of the same bird (case FS415/23). The positive Stone-curlew, an adult female, was found alive in an urban area in northern Gran Canaria (Table A1). Due to the severe lesions on its pelvic limbs consistent with advanced avian poxvirus infection, combined with the overall poor clinical condition of the bird, euthanasia was performed shortly after admission to the Wildlife Recovery Center of Tafira. The avian poxvirus infection was later confirmed by PCR and histopathological analysis [29]. In addition, an *Aspergillus fumigatus* infection was detected and confirmed by PCR [29].

**Figure 2:**
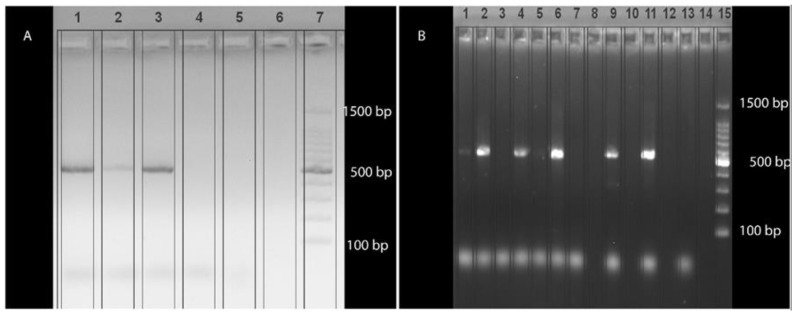
Agarose gel (2%) electrophoresis of conventional semi-nested PCR products targeting the Haemosporidian *cytochrome b* gene. (**A**). Amplified products of the expected size (≈522 bp) are detected in lanes 1 and 2, corresponding to two replicates of the pooled tissue sample of case FS415/23. Lane 3 represents the commercial positive control (*Plasmodium falciparum*), lane 4 the negative control, lanes 5 and 6 are empty, and lane 7 contains the 100 bp DNA ladder. (**B**). Amplicons of the expected size (≈522 bp) are observed in liver (lane 2), lung (lane 4), kidney (lane 6), and skin (lane 9) samples, as well as in the commercial positive control (lane 11). Lane 13 corresponds to the negative control, and lane 15 contains the 100-bp DNA ladder.

Amplicons obtained from the semi-nested PCR revealed a band of the expected size (approximately 522 bp) (Figure 2). Further sequencing of the positive samples produced a uniform consensus sequence of approximately 476 bp (primers excluded). This sequence clustered within the genus *Leucocytozoon*, showing 100% nucleotide identity and query coverage with multiple *Leucocytozoon* spp. sequences from diverse avian hosts across different geographic regions, and has been deposited in GenBank under accession number PX377274. The same skin sample had previously tested positive for *Avipoxvirus* (AVP) and *Aspergillus fumigatus* DNA by PCR, as reported in an earlier study [29].

### 3.3. Phylogenetic Analysis

**Figure 3:**
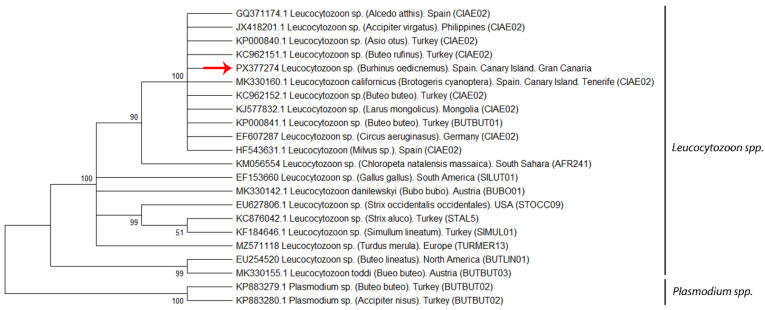
Phylogenetic analysis of positive case sequence (arrow) (GenBank accession number: PX377274), obtained from liver, kidney, and skin samples, based on partial mitochondrial cytochrome b sequences from various host species (indicated in brackets next to the *Leucocytozoon* and *Plasmodium* species names). Lineages from previous studies are labeled in parentheses. Evolutionary history was inferred using the Maximum Likelihood method [42]. The bootstrap consensus tree, inferred from 1000 replicates [42], represents the evolutionary relationship of the taxa analyzed, with branches supported in less than 50% of replicates collapsed. The percentage of replicate trees in which associated taxa clustered together is shown next to the branches [42]. The initial tree for the heuristic search was selected as the one with the superior log-likelihood between a Neighbor-Joining (NJ) tree [43] and a Maximum Parsimony (MP) tree. The NJ tree was generated using a matrix of pairwise distances computed with the p-distance [44]. The rate model allowed for 59.10% of sites to be evolutionarily invariable (I). The analysis included 22 coding nucleotide sequences, incorporating 1st, 2nd, 3rd, and non-coding positions, with 526 positions in the final dataset. Evolutionary analyses were conducted in MEGA12 [41] utilizing up to 3 parallel computing threads.

The mitochondrial *cytochrome b* gene sequence obtained from the Haemosporidia-positive Stone-curlew was analyzed to assess its phylogenetic relationships. This sequence (GenBank accession ID: PX377274) was included in a phylogenetic analysis with 21 other haemosporidian sequences retrieved from GenBank, comprising 19 *Leucocytozoon* spp. and two *Plasmodium* spp. sequences used as outgroups. The phylogenetic tree was constructed using the T92 + I substitution model (Figure 3). Our sequence clustered closely with the CIAE02 linage reported from several countries, including Turkey [KP000840, KC962151, KC962152, KP000841*(BUTBUT01 linage)], Spain (GQ371174, HF543631, MK330160), the Philippines (JX418201), Mongolia (KJ577832), and Germany (EF607287), showing 100% nucleotide identity in BLASTn (BLAST+ 2.17.0) searches and strong phylogenetic support in maximum likelihood analyses (bootstrap = 100%) (Figure 3). Lineage assignment was determined by comparison with publicly available sequences in the GenBank and MalAvi databases (mbio-serv2.mbioekol.lu.se/Malavi) [18].

### 3.4. Histopathological Findings

**Figure 4:**
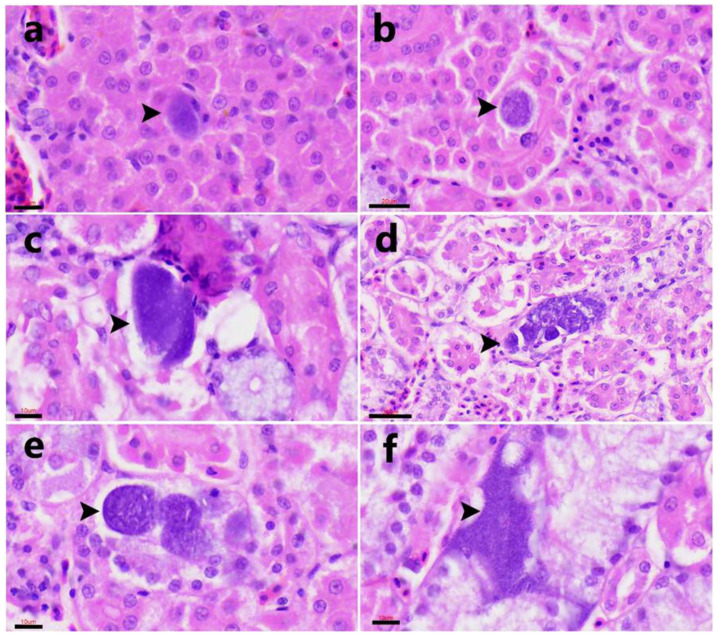
Case FS415/23. Exo-erythrocytic tissue stages of *Leucocytozoon* sp. parasites (arrowheads) in hematoxylin-eosin (H&E) stained sections. (**a**) Early hepatic meront (scale bars =10 µm). (**b**–**f**) Meronts of irregular shape and size were detected within the epithelial cells of renal tubules. (**b**). A small meront in a renal epithelial cell (scale bars = 20 µm). (**c**). A more mature meront packed with merozoites, occupying the entire intracellular space and causing protrusion into the tubular lumen (scale bar = 30 µm). (**d**,**e**) Developing megalomeronts in the kidney ((**d**). Scale bar = 30 µm). (**f**). Mature meront within a renal epithelial cell. Scale bars =10 µm, unless otherwise indicated.

In histological sections, exo-erythrocytic stages consistent with Haemosporidia infection were identified in three of the four PCR-positive tissues. Meront-like structures at various maturation stages were observed within hepatocytes (Figure 4a) and within renal epithelial cells (Figure 4b–f). These parasitic stages were characterized by rounded (10–20 µm in diameter) to oval (35–70 µm in length) structures containing multiple basophilic nuclei. No inflammatory infiltrate, necrosis, or other tissue reaction was evident in any of the examined tissues.

**Figure 5:**
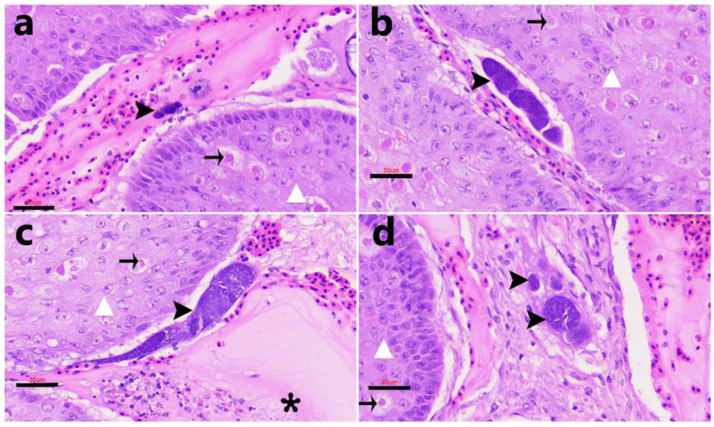
Case FS415/23. Exo-erythrocytic stages of *Leucocytozoon* sp. in hematoxylin-eosin (H&E)-stained skin sections. The skin shows severe epidermal hyperplasia (white triangle) with characteristic *Avipoxvirus* eosinophilic intracytoplasmic inclusion bodies (Bollinger bodies) (arrow). (**a**). Intravascular meront (arrowhead) (**b**). Incipient megalomeront (arrowhead) within a dermal vessel. (**c**). Incipient megalomeront (arrowhead) within a dermal vessel, with multiple free merozoites surrounded by inflammatory cells (asterisk). (**d**). Incipient megalomeronts within the dermis. Scale bars = 30 µm.

**Figure 6:**
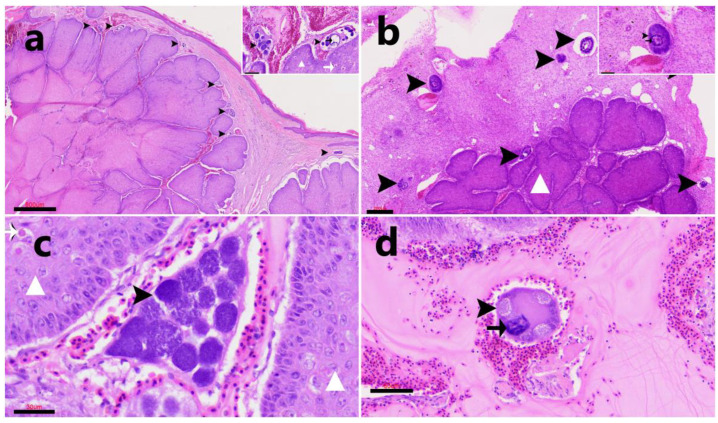
Case FS415/23. Histological sections (Hematoxylin & Eosin stain) of avian skin showing exo-erythrocytic stages of *Leucocytozoon* sp. and concurrent *Avipoxvirus* infection characterized by epidermal hyperplasia (white triangle); Bollinger bodies (white arrow). (**a**). Overview of the dermis and epidermis showing multiple large parasitic stages (arrowheads) consistent with *Leucocytozoon* megalomeronts beneath marked epidermal hyperplasia. Scale bar = 800 µm. Inset: higher magnification of a blood vessel containing developing megalomeronts (arrowhead) with multiple internal nuclei (merozoites) and hypertrophied host cell nuclei (arrow). Scale bar = 70 µm. (**b**). Multiple incipient megalomeronts within or adjacent to dermal vessels (arrowheads). Scale bar = 200 µm. Inset: developing megalomeront (arrowheads) with internal nuclei (merozoites) and hypertrophied host cell nuclei (arrow). Scale bar = 60 µm. (**c**). Mature meront (arrowhead) in a dermal vessel. Scale bar = 30 µm. (**d**). Vascular-associated megalomeront (arrowheads) with internal merozoites and a hypertrophied host cell nucleus (arrow). Scale bar = 60 µm.

In the skin, mature meronts (Figure 5) and numerous megalomeronts (Figure 6) exhibiting considerable morphological variability were observed within the dermis. These exo-erythrocytic stages were mostly located inside capillaries. The megalomeronts appeared as large, round to oval structures—often exceeding 50 µm in diameter—with a thick eosinophilic wall that frequently enclosed an enlarged host cell nucleus. The internal content consisted of numerous cytomeres arranged within a granular to finely vacuolated basophilic cytoplasm, containing multiple nuclei at various stages of development. The affected skin also exhibited marked epidermal hyperplasia, ballooning degeneration, and intracytoplasmic eosinophilic inclusion bodies (Bollinger bodies) consistent with *Avipoxvirus* infection, as well as fungal hyphae morphologically compatible with *Aspergillus* spp. in the necrotic crusts and superficial dermis. Despite the presence of these concurrent infections, no prominent inflammatory reaction was observed surrounding the haemosporidian stages. On rare occasions, small, round, basophilic structures consistent with free merozoites were observed within the vascular plasma, apparently representing stages preceding erythrocytic invasion. These were surrounded by a localized inflammatory response composed predominantly of macrophages and heterophils (Figure 5c).

**Figure 7:**
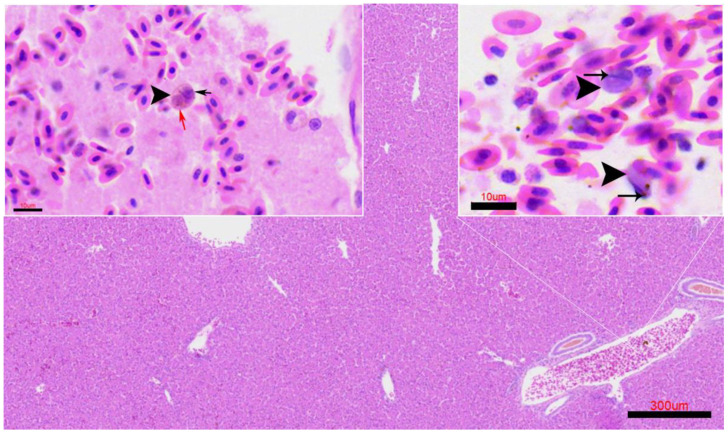
Case FS415/23. Intra-erythrocytic-like stage of *Leucocytozoon* sp. (arrowheads) observed in hematoxylin-eosin (H&E)- stained liver sections. Scale bar = 300 µm. Upper left inset: liver vessel showing a parasite nucleolus (red arrow). Upper right inset: blood vessel containing structures compatible with the intra-erythrocytic form of *Leucocytozoon* sp.; host cell nuclei indicated (black arrow). Inset scale bar = 10 µm.

Occasionally, structures compatible with intra-erythrocytic stages of *Leucocytozoon* sp. were observed within the blood vessels of the liver (Figure 7) and kidney. The infected circulating cells appeared enlarged and distorted, containing eccentrically placed basophilic nuclei and cytoplasmic inclusions consistent with developing gametocytes.

## 4. Discussion

This study represents the first molecular and histopathological confirmation of *Leucocytozoon* sp. infection in the Stone-curlew. The detection of the only *Leucocytozoon*-positive Stone-curlew in Gran Canaria is consistent with the distribution and population structure of the species in the archipelago. Gran Canaria holds one of the largest and most stable populations of *Burhinus oedicnemus distinctus* in the western islands, with approximately 250 breeding pairs [35], which increases the likelihood of encountering infected individuals compared with islands where the species is less abundant. In our study, samples were geographically distributed across the main islands where the species occurs, and thus not restricted to a single locality. However, population sizes differ markedly between islands, and this must be considered when interpreting the spatial pattern of infection.

The identification of parasite DNA in several organs, including the skin, liver, kidney, and lungs, together with the observation of characteristic exo-erythrocytic stages, provides clear evidence of a systemic infection and confirms the susceptibility of this species to haemosporidian parasites. To date, information on blood parasite diversity within the genus *Burhinus*, and particularly in the Eurasian Stone-curlew, remains scarce. A notable exception is the report of *Haemoproteus burhinus* in the Saharan subspecies *Burhinus oedicnemus saharae* from Iraq, described by Mohammad (1998) [45], which was characterized by hypertrophied infected erythrocytes and lateral displacement of the host cell nucleus in microgametocytes. Taken together, these findings broaden our understanding of haemosporidian diversity and host–parasite associations within the family Burhinidae.

Moreover, the detection of *Leucocytozoon* sp. in this species demonstrates the capacity of this parasite to infect Charadriiform hosts, a group in which such infections are infrequently reported. According to the MalAvi database, more than 5300 haemosporidian lineages have been identified to date, but only 67 have been associated with Charadriiformes. Most of these belong to *Haemoproteus* (23 lineages) and *Plasmodium* (39 lineages), while *Leucocytozoon* accounts for merely five lineages, emphasizing the rarity of such infections in this avian order [18].

Molecular characterization based on *cytochrome b* sequencing and comparison with the MalAvi database confirmed that the detected *Leucocytozoon* isolate belongs to the lineage CIAE02. According to MalAvi records, this lineage exhibits an exceptionally broad host and geographical range, having been reported from more than ten avian orders, including Falconiformes, Strigiformes, Gruiformes, and Charadriiformes, and across four continents: Europe, Asia, Africa, and North America [46,47,48,49,50,51,52]. It has been previously reported in raptors such as Eleonora’s Falcon (*Falco eleonorae*) in Alegranza Island (Canary Islands) and Black Kite (*Milvus migrans*) in southern Spain [47,53].

Although CIAE02 is most frequently associated with raptors (Accipitridae and Falconidae), its occurrence in hosts occupying diverse ecological niches, such as rails (*Crex crex*), gulls (*Larus* spp.), woodpeckers (*Dryocopus martius*), and even parrots (*Brotogeris cyanoptera*), indicates either an unusually generalist parasite with low host specificity or a complex of closely related cryptic lineages that cannot yet be genetically distinguished. Detection of this lineage in the Stone-curlew further extends its known host spectrum to the family Burhinidae and suggests possible ecological overlap in vector exposure or occasional host switching among sympatric bird species. Further molecular and morphological investigations are required to clarify the taxonomic boundaries of CIAE02 and to better understand host–parasite associations and transmission dynamics of *Leucocytozoon* in Charadriiform birds.

The identification of exo-erythrocytic meronts and megalomeronts in the liver and kidney is consistent with the typical life cycle of *Leucocytozoon* spp., in which asexual replication occurs in multiple tissues before gametocyte formation in circulating blood cells. The histological detection of meronts and megalomerts in the liver and kidneys agrees with previous descriptions regarding their morphology and localization in other avian hosts [4,6,9,10,54,55,56,57,58,59,60,61]. However, the presence of megalomeronts in the skin represents an uncommon finding, as their development in dermal tissue has been rarely documented [9]. Their localization within dermal capillaries may indicate broader tissue tropism or atypical development, potentially influenced by host species or concurrent infections [3,9]. In this individual, *Avipoxvirus* lesions and *Aspergillus fumigatus* infection were present in the skin [28,29], potentially altering local tissue conditions and facilitating aberrant parasite development.

Although no previous reports of coinfection involving these specific pathogens have been found, several studies have documented concurrent *Plasmodium* and *Avipoxvirus* infections in island bird species, where such associations have been linked to population declines and even local extinctions [62,63,64,65]. The frequent coexistence of these pathogens is often explained by the overlap of their insect vectors [3,63,66]. However, in the present case, the vectors involved are not generally shared between both pathogens [1,67,68,69]. While all hemosporidian parasites are transmitted by Diptera, each genus is typically associated with a specific dipteran family [1,7,66]. Only *Leucocytozoon caulleryi* is known to be transmitted by biting midges (*Culicoides* spp.) [1], whereas all other *Leucocytozoon* species are vectored by black flies (*Simuliidae*). Their ecological distribution and microhabitat preference play a key role in shaping parasite transmission dynamics and can impose constraints on the parasite’s host range [70,71]. Although many ornithophilic black fly species do not exhibit strict host specificity, they frequently show strong fidelity to particular habitat niches, such as foraging or breeding predominantly in the upper forest canopy, which in turn influences which bird species they are most likely to encounter and infect [72,73].

Although black flies generally require humid environments with continuous flowing water for larval development, the ecological conditions in the Canary Islands are comparatively challenging due to the limited and patchy distribution of permanent freshwater streams. Even under these constraints, five Simuliidae species (*Simulium guimari*, *S. intermedium*, *S. paraloutetense*, *S. ruficorne*, and *S. velutinum*) are currently recognized in the archipelago, with *S. guimari* consistently recorded as the most abundant and widespread. Species richness also varies among islands; notably, Gran Canaria and La Gomera each support all five taxa [74]. The persistence of these species indicates that even small or seasonal water sources, including ravines, remnants of laurel forest, and human-created water channels, provide sufficient habitat to maintain black fly populations that may interact with local avifauna and facilitate the transmission of *Leucocytozoon* parasites [53,75,76,77].

Common pathological alterations associated with leucocytozoonosis include tissue damage and inflammatory reactions related to the presence of megalomeronts, which represent the final stage of exo-erythrocytic development. In addition, vascular congestion and focal tissue degeneration may occur because of intravascular accumulations of intraerythrocytic stages, potentially impairing blood flow and oxygen delivery, thereby contributing to local hypoxia and organ dysfunction [3,9]. Notably, no marked inflammatory response was detected around most parasitic stages, despite concurrent *Avipoxvirus* and *Aspergillus fumigatus* infections, suggesting a subacute or chronic infection phase, or possibly a degree of host adaptation. In contrast, focal inflammatory responses were observed, characterized by leukocytosis associated with free merozoites within dermal blood vessels, indicating that rupture of parasitic stages and antigen release can locally activate the host immune system. Structures resembling intra-erythrocytic stages were rarely observed in the liver, kidneys, and lungs, indicating a predominance of exo-erythrocytic development and suggesting a chronic or subclinical infection. These findings align with the low parasitaemia typically reported in *Leucocytozoon* infections [3,7,9,10].

The low infection prevalence detected in our study (2%) contrasts with previous surveys in the archipelago based on blood smears. For instance, Bodawatta et al. (2020) [78] reported a prevalence of 15% in passerine species, while the survey conducted in Tenerife documented 13.4% infection, primarily in Passeriformes but including one positive *Accipiter nisus* carrying the same lineage identified in our material (CIAE02) [75]. Spurgin et al. (2021) [65] recorded a prevalence of 9.6% in Berthelot’s pipit (*Anthus berthelotii*) in 2006, which declined to 0% in 2009. Additionally, a prevalence of 0.5% was reported for Eleonora’s falcon, also infected with the same lineage found in our study [53]. These differences highlight the influence of sampling method, species composition, and temporal variation on observed prevalence patterns and underscore the need for integrating both blood-based and tissue-based approaches in future haemosporidian surveys.

Most studies on avian haemosporidians rely on blood samples from live birds, which enable the detection of circulating gametocytes and estimation of parasitemia levels. However, recent research has demonstrated that these parasites can persist in internal organs and may not always be detectable in peripheral blood [5,79]. More recently, Heaver et al. (2025) [80] detected *Plasmodium* and *Haemoproteus* DNA in 13.5% of over 850 wild birds examined postmortem in Great Britain and confirmed exoerythrocytic parasite stages and lesions consistent with avian malaria in several cases. Collectively, these studies highlight that postmortem tissue screening can reveal latent or tissue-restricted infections and provide histopathological context that is unavailable from blood samples alone. Nevertheless, factors such as postmortem degradation, uneven parasite distribution, and differences in tissue tropism may influence detection sensitivity. Consequently, infection data derived from tissues should be interpreted with caution and not directly compared with blood-based prevalence estimates. Instead, both approaches are best viewed as complementary, together offering a more comprehensive understanding of haemosporidian diversity, distribution, and host–parasite dynamics in wild bird populations.

In the present study, blood smears were not available, and parasite detection relied exclusively on molecular screening of tissues and histopathological examination. Although the lack of blood films limits the morphological characterization of blood stages and precludes quantification of parasitemia, the combination of molecular and histological evidence still provides important insight into the infection process. The detection of parasite DNA in multiple organs, together with the presence of exo-erythrocytic stages and rare gametocyte-like forms, suggests that parasite development was initiated but possibly not completed. The limited inflammatory reaction observed in association with these lesions further supports the hypothesis of an early or abortive infection rather than a fully established systemic parasitemia.

In non-adapted hosts, *Leucocytozoon* spp. may undergo incomplete development, resulting in the formation of tissue stages that fail to mature into gametocytes, while still inducing marked histopathological alterations [3,9,62]. This phenomenon reflects a mismatch between parasite developmental requirements and host cellular or immunological conditions. Accordingly, the dermal megalomeronts observed in this Stone-curlew likely represent abortive development in a non-evolutionarily adapted host, a scenario previously reported in *Leucocytozoon simondi* infections in non-adapted waterfowl [9].

From an epidemiological perspective, the occurrence of *Leucocytozoon* in a Stone-curlew from the Canary Islands raises questions about the distribution of competent vectors and the potential impact on local bird populations. The presence of suitable vectors in the archipelago, combined with the insular isolation and small population size of the endemic subspecies (*B. o. insularum* and *B. o. distinctus*), may pose a potential conservation concern, as even low-prevalence infections could contribute to morbidity or reduced fitness in vulnerable populations. Previous studies in island birds have demonstrated that limited genetic diversity and restricted dispersal can amplify the effects of infectious diseases on population dynamics [3,5,81].

## 5. Conclusions

The present findings expand the known host range of *Leucocytozoon* and emphasize the value of combining molecular screening with histopathology to detect low-parasitemia or cryptic infections, particularly in non-passerine hosts. Integrative taxonomic approaches that incorporate morphological, molecular, and vector data are essential to determine whether this lineage represents a distinct species or an intraspecific variant. The histological evidence of atypical tissue tropism and possible abortive development observed in this Stone-curlew suggests that this host–parasite relationship may represent a recent or accidental host shift rather than a coevolved association.

## Data Availability

The original contributions presented in this study are included in the article.

## Abbreviations

The following abbreviations are used in this manuscript:

IUSA	University Institute of Animal Health and Food Safety
ULPGC	University of Las Palmas de Gran Canaria

## Appendix A

**Table A1:** Data from 47 stone-curlews (*Burhinus oedicnemus*) included in the present study (2020–2024), comprising biological information (age and sex), island of origin and recorded coordinates (latitude and longitude), condition (dead or alive) at the time of discovery, state of decomposition at necropsy, body condition, and the samples tested for Haemosporidian DNA detection.

ID	Age	Sex	FL	GC	FD	SS	DC	BC	Sample Tested	Haemosporidian PCR Results
SA324/20	A	U	L	29°6′35.86″ N, 13°33′21.72″ W	30/07/2020	D	3	1	Pool*	-
SA131/21	J	F	L	29°14′51.89″ N, 13°30′44.34″ W *	08/08/2020	D	2	1	Pool*	-
SA132/21	A	F	L	28°56′57.90″ N, 13°46′16.37″ W *	03/09/2020	D	3	1	Pool*	-
SA133/21	A	F	L	29°3′49.73″ N, 13°33′55.21″ W *	24/11/2020	D	3	1	Pool*	-
SA134/21	J	M	L	28°56′37.94″ N, 13°46′47.15″ W *	04/12/2020	D	2	3	Pool*	-
SA255/21	J	M	F	28°30′21.84″ N, 13°53′2.39″ W	20/06/2018	D	3	3	Pool*	-
SA334/21	A	M	GC	28°6′13.79″ N, 15°41′58.93″ W *	22/04/2021	D	2	2	Pool*	-
SA359/21	A	U	T	29°4′34.42″ N, 13°31′19.56″ W	29/04/2021	D	3	3	Pool*	-
SA447/21	J	U	L	29°5′31.98″ N, 13°37′24.62″ W	24/05/2021	D	3	1	Pool*	-
SA485/21	A	F	L	28°59′35.28″ N, 13°36′18.00″ W	08/04/2021	D	2	3	Pool*	-
SA518/21	J	M	L	28°59′35.28″ N, 13°36′18.00″ W	06/04/2021	D	2	2	Pool*	-
SA597/21	J	M	L	28°56′55.55″ N, 13°36′22.06″ W	21/12/2018	D	2	2	Pool*	-
SA849/21	J	U	GC	28°5′1.74″ N, 15°27′18.20″ W	27/08/2021	D	3	3	Pool*	-
SA870/21	J	M	GC	28°6′38.85″ N, 15°28′21.07″ W *	U	D	2	1	Pool*	-
SA875/21	J	F	L	28°57′55.56″ N, 13°32′52.69″ W *	07/06/2021	D	2	U	Pool*	-
SA876/21	A	M	L	28°57′27.70″ N, 13°34′14.15″ W	08/07/2021	D	2	2	Pool*	-
SA877/21	J	M	L	29°7′52.43″ N, 13°27′45.56″ W	29/06/2021	D	3	3	Pool*	-
SA994/21	J	M	L	29°12′0.67″ N, 13°27′9.54″ W	31/08/2021	D	3	3	Pool*	-
SA1133/21	A	M	T	28°28′29.84″ N, 16°20′53.60″ W *	22/0/2021	D	2	2	Pool*	-
SA1156/21	A	F	F	28°36′2.63″ N, 13°55′19.53″ W *	25/10/2021	D	3	2	Pool*	-
SA1262/21	A	F	T	28°6′37.96″ N, 16°43′51.44″ W *	04/11/2021	D	2	3	Pool*	-
SA1295/21	J	F	GC	28°4′8.43″ N, 15°27′13.40″ W	U	D	3	2	Pool*	-
SA1296/21	J	F	GC	28°4′8.43″ N, 15°27′13.40″ W	18/09/2021	D	2	1	Pool*	-
SA1297/21	A	F	GC	28°4′8.43″ N, 15°27′13.40″ W	28/09/2021	D	2	3	Pool*	-
SA1298/21	J	F	GC	28°4′8.43″ N, 15°27′13.40″ W	U	D	1	3	Pool*	-
SA1411/21	A	F	T	28°28′37.20″ N, 16°19′15.71″ W	30/11/2021	D	3	2	Pool*	-
SA1417/21	A	F	T	28°28′37.20″ N, 16°19′15.71″ W	07/09/2021	D	2	3	Pool*	-
SA1438/21	A	F	GC	28°4′8.43″ N, 15°27′13.40″ W	12/11/2021	D	2	2	Pool*	-
SA1439/21	A	M	GC	28°4′8.43″ N, 15°27′13.40″ W	U	D	2	2	Pool*	-
SA1505/21	A	U	L	28°58′44.49″ N, 13°31′4.29″ W	26/11/2021	D	2	1	Pool*	-
SA1506/21	A	F	L	29°3′50.86″ N, 13°33′20.79″ W *	10/11/2021	D	3	1	Pool*	-
SA061/22	A	M	L	28°58′22.42″ N, 13°35′7.82″ W	10/12/2021	D	3	2	Lung	-
SA301/22	A	F	L	29°6′15.60″ N, 13°28′21.07″ W	27/02/2022	D	3	2	Pool*	-
SA1246/22	C	U	GC	28°4′8.43″ N, 15°27′13.40″ W	05/05/2022	D	3	3	Pool*	-
SA1267/22	A	F	GC	28°7′12.30″ N, 15°28′56.82″ W *	31/05/2022	A	3	2	Pool*	-
SA1269/22	J	U	GC	28°6′32.11″ N, 15°27′51.01″ W *	08/08/2022	A	2	U	Pool*	-
SA1277/22	U	M	GC	28°7′11.14″ N, 15°28′37.43″ W *	U	D		2	Pool*	-
SA1283/22	J	M	GC	27°55′14.36″ N, 15°24′50.89″ W	26/06/2022	A	2	2	Pool*	-
SA1365/22	A	F	H	27°42′36.42″ N, 17°59′55.14″ W *	U	D	3	2	Pool*	-
SA1508/22	J	M	GC	28°6′41.54″ N, 15°29′26.38″ W *	28/09/2022	D	2	3	Pool*	-
SA1642/22	A	M	L	28°59′51.72″ N, 13°36′13.03″ W *	03/10/2022	D	2	1	Pool*	-
FS229/23	J	U	L	28°54′0.03″ N, 13°50′1.64″ W	12/02/2023	D	2	1	Pool*	-
FS415/23	A	F	GC	28°6′10.56″ N, 15°41′59.18″ W *	05/05/2023	A	1	1	Pool*, Liver, kidney, lung, skin	*Leucocytozoon*. CIAE02 linage.
FS0453/23	A	M	L	29°5′4.78″ N, 13°39′55.37″ W	08/04/2023	D	2	2	Pool*	-
FS0546/23	A	F	GC	28°7′6.68″ N, 15°31′21.48″ W *	U	D	2	2	Pool*	-
FS499/24	A	M	GC	28°6′27.51″ N, 15°35′0.19″ W *	03/09/24	A	2	1	Pool*	-
FS601/24	A	M	GC	28°3′53.96″ N, 15°27′44.07″ W	25/06/24	A	2	1	Pool*	-

Notes: Pool* = Liver, kidney, lung; FD (finding date); FL (finding location: GC = Gran Canaria; T = Tenerife; F = Fuerteventura; L = Lanzarote); GC (Geographical coordinates, Latitude/Longitude (* Approximate coordinates by locality); S (status: A = alive; D = dead); DC (decomposition code); DC (1 = very fresh, 2 = fresh, 3 = incipient decomposition); BC (body condition); BC (1 = cachexia, 2 = thin (slim), 3 = good body condition, U = undetermined); Sex (F = female, M = male, U = undetermined), Age (A = adult, J = juvenile, C = chick, U = undetermined); Underlined = Positive individual.
